# Supplementary material for: From sequence to enzyme mechanism using multi-label machine learning
Source: BMC Bioinformatics. 2014 May 19;15:150. doi: 10.1186/1471-2105-15-150 (PMC4229970; doi:10.1186/1471-2105-15-150)
Supplement: Additional file 2 — Java code of ml2db. Additional file ml2db_code.tar.gz contains the Java source code to run the multi-label machine learning experiments and save the results to database. The code’s Javadoc is included. [file 1471-2105-15-150-S2.zip › additional file 2/ml2db/ml2db/doc/main/ml2db/al/utility/globaldistance/GlobalPositionStore.html]

GlobalPositionStore


JavaScript is disabled on your browser.


- Overview
- Package
- Class
- Use
- Tree
- Deprecated
- Index
- Help

- Prev Class
- Next Class

- Frames
- No Frames

- All Classes

- Summary:
- Nested |
- Field |
- Constr |
- Method

- Detail:
- Field |
- Constr |
- Method


main.ml2db.al.utility.globaldistance

## Class GlobalPositionStore

- java.lang.Object
- - main.ml2db.al.utility.globaldistance.GlobalPositionStore

- ---

    

  ```
  public class GlobalPositionStore
  extends java.lang.Object
  ```

  Stores (and updates) a map containing as key one (kind of/set of attributes)
  instance and as value the number representing the distance between the
  instance and a set of other labelled or unlabelled instances.

  Version:
  :   19 Jul 2011

  Author:
  :   Luna De Ferrari ldf(at)st-andrews.ac.uk

- - ### Field Summary

    Fields

    | Modifier and Type | Field and Description |
    | --- | --- |
    | `static int` | `LABELLED_ROLE` |
    | `static int` | `UNLABELLED_ROLE` |
  - ### Constructor Summary

    Constructors

    Constructor and Description

    `GlobalPositionStore(int hashInitialSize,
    GlobalDistanceMetric positioningMetric)`
  - ### Method Summary

    Methods

    | Modifier and Type | Method and Description |
    | --- | --- |
    | `void` | `addDistances(java.lang.String instanceAttributes, java.lang.Double distance, int labellingRole)` Add an instance as sparse instance attributes string: {attribute index attribute value, ...} eg. |
    | `java.lang.Double` | `getDistances(java.lang.String instanceAttributes, int labellingRole)` Find the distance of an instance to all labelled instances. |
    | `java.util.HashMap<java.lang.String,java.lang.Double>` | `getGlobPositionStore(int labellingRole)` |
    | `GlobalDistanceMetric` | `getPositioningMetric()` |
    | `void` | `setTest(boolean test)` |
    | `void` | `updateDistances(weka.core.Instance instanceLabelled)` |

    - ### Methods inherited from class java.lang.Object

      `equals, getClass, hashCode, notify, notifyAll, toString, wait, wait, wait`

- - ### Field Detail


    - #### LABELLED\_ROLE

      ```
      public static final int LABELLED_ROLE
      ```

      See Also:
      :   Constant Field Values


    - #### UNLABELLED\_ROLE

      ```
      public static final int UNLABELLED_ROLE
      ```

      See Also:
      :   Constant Field Values
  - ### Constructor Detail


    - #### GlobalPositionStore

      ```
      public GlobalPositionStore(int hashInitialSize,
                         GlobalDistanceMetric positioningMetric)
      ```
  - ### Method Detail


    - #### addDistances

      ```
      public void addDistances(java.lang.String instanceAttributes,
                      java.lang.Double distance,
                      int labellingRole)
      ```

      Add an instance as sparse instance attributes string: {attribute index
      attribute value, ...} eg. {8 1,9 1} and its cumulative distance to all
      currently labelled instances.

      Parameters:
      :   `instanceAttributes` -
      :   `distance` -
      :   `role` - whether this distance should be stored for the labelled or
          unlabelled set


    - #### getDistances

      ```
      public java.lang.Double getDistances(java.lang.String instanceAttributes,
                                  int labellingRole)
      ```

      Find the distance of an instance to all labelled instances. The instance
      should be represented as sparse instance attributes string: {attribute
      index attribute value, ...} eg. {8 1,9 1}.

      Parameters:
      :   `an` - instance represented as sparse instance attributes string
      :   `role` - whether the requested distance is to the labelled or
          unlabelled set

      Returns:
      :   the distance of an instance to all labelled instances.


    - #### getGlobPositionStore

      ```
      public java.util.HashMap<java.lang.String,java.lang.Double> getGlobPositionStore(int labellingRole)
      ```


    - #### getPositioningMetric

      ```
      public GlobalDistanceMetric getPositioningMetric()
      ```


    - #### setTest

      ```
      public void setTest(boolean test)
      ```

      Parameters:
      :   `deserialized` - m\_deserialized to set


    - #### updateDistances

      ```
      public void updateDistances(weka.core.Instance instanceLabelled)
      ```


- Overview
- Package
- Class
- Use
- Tree
- Deprecated
- Index
- Help

- Prev Class
- Next Class

- Frames
- No Frames

- All Classes

- Summary:
- Nested |
- Field |
- Constr |
- Method

- Detail:
- Field |
- Constr |
- Method
